# DNACPR Documentation Audit on Old Age Psychiatry Wards in Aneurin Bevan University Health Board (ABUHB) in South Wales

**DOI:** 10.1192/bjo.2025.10663

**Published:** 2025-06-20

**Authors:** Amr Romeh, Gianluca Mosaico, Mohammed Elhusseiny, Peter Boyle, Tanbir Kalam

**Affiliations:** Aneurin Bevan University Health Board, Caerphilly, United Kingdom

## Abstract

**Aims:** DNACPR All-Wales policy for adults was launched in 2015 by the deputy minister for Health. It was revised in 2017, 2020, 2022 and 2024. DNACPR decisions should be clearly recorded and communicated between health professionals.

This audit aims at assessing the quality of DNACPR on older adult psychiatric wards in ABUHB to identify any deficits and to improve the quality of documentation in the future.

**Methods:** Inpatients’ notes from all four old age psychiatry wards in ABUHB were examined; patients with DNACPR were identified. To gather data, we created an audit tool based on all-Wales policy.

**Results:** 24 DNACPR decision forms were identified.

“Decision date” was missed in 2 forms.

DNACPR decision was not clearly “signed with date and time” in 1 form.

In 10 forms, decision was not “documented in clinical notes” because the decision was made by another team and their notes were not available to examine.

“Discussion with patient” only took place in 9 forms while “discussion with IMCA/attorney or family/carers” took place in 20 forms.

“Patient demographic details” were recorded in all forms but with some errors; one patient’s name was incorrectly spelt and one patient had missing details.

For “reason of decision”, 1 was “not in the best interest, natural anticipated and accepted death and patient refused CPR”, 1 was “patient refused CPR” and 1 had no stated reason. The remaining 21 forms stated “not in the best interest”.

For “signatures in section 5 and section 6” where section 5 is for “the health care professional completing the form” while section 6 is for “the senior responsible clinician”. The signatures were appropriate in 17 forms. 7 forms were not countersigned in section 6; 5 of these are signed in section 5 by a consultant while 1 is signed by a junior doctor and 1 is signed by a registered nurse.

Different versions of the DNACPR form were being used in some instances.

**Conclusion:** Identified deficits represent deficient forms which could lead to inappropriate CPR attempts. This could lead to physical and emotional distress and possible litigation.

To avoid this, our audit concludes that DNACPR forms must be completed thoroughly and that a unified version of DNACPR form is to be used. It is also good practice to document the DNACPR discussion in the clinical notes of the patients.

As secondary outcomes, we recommended to add DNACPR status and treatment escalation plan status to our ward round proforma.

